# Biomechanical evaluation of a novel intervertebral disc repair technique for large box-shaped ruptures

**DOI:** 10.3389/fbioe.2023.1104015

**Published:** 2023-02-08

**Authors:** Mao-Dan Nie, Ze-Bin Huang, Ning-Ze Zhang, Ling-Jie Fu, Cheng-Kung Cheng

**Affiliations:** ^1^ School of Biomedical Engineering and Engineering Research Center of Digital Medicine of the Ministry of Education, Shanghai Jiao Tong University, Shanghai, China; ^2^ Department of Spine Surgery, First Affiliated Hospital of Second Military Medical University, Shanghai, China; ^3^ Beijing Advanced Innovation Centre for Biomedical Engineering, School of Biological Science and Medical Engineering, Beihang University, Beijing, China; ^4^ Shanghai Key Laboratory of Orthopaedic Implants, Department of Orthopaedic Surgery, Shanghai Ninth People’s Hospital, Shanghai Jiao Tong University School of Medicine, Shanghai, China

**Keywords:** herniated disc, annular rupture, annular patch, material properties, biomechanical evaluation

## Abstract

**Objective:** The purpose of this study was to analyze the feasibility of repairing a ruptured intervertebral disc using a patch secured to the inner surface of the annulus fibrosus (AF). Different material properties and geometries for the patch were evaluated.

**Methods:** Using finite element analysis, this study created a large box-shaped rupture in the posterior-lateral region of the AF and then repaired it with a circular and square inner patch. The elastic modulus of the patches ranged from 1 to 50 MPa to determine the effect on the nucleus pulposus (NP) pressure, vertical displacement, disc bulge, AF stress, segmental range of motion (ROM), patch stress, and suture stress. The results were compared against the intact spine to determine the most suitable shape and properties for the repair patch.

**Results:** The intervertebral height and ROM of the repaired lumbar spine was similar to the intact spine and was independent of the patch material properties and geometry. The patches with a modulus of 2–3 MPa resulted in an NP pressure and AF stresses closest to the healthy disc, and produced minimal contact pressure on the cleft surfaces and minimal stress on the suture and patch of all models. Circular patches caused lower NP pressure, AF stress and patch stress than the square patch, but also caused greater stress on the suture.

**Conclusion:** A circular patch with an elastic modulus of 2–3 MPa secured to the inner region of the ruptured annulus fibrosus was able to immediately close the rupture and maintain an NP pressure and AF stress similar to the intact intervertebral disc. This patch had the lowest risk of complications and produced the greatest restorative effect of all patches simulated in this study.

## 1 Introduction

Intervertebral discs (IVDs) cushion compression forces on the spine whilst providing considerable flexibility. Disc herniation manifests as a protrusion of the soft inner nucleus pulposus (NP) through the outer annulus fibrosus (AF), which can ultimately compromise the structural integrity of the disc. The protrusion can also compress nerves in the region, with patients often complaining of pain and numbness in the back and lower extremities. Discectomy is an effective treatment to relieve the symptoms of herniated discs, but there is a risk of re-herniation due to original annular rupture (Carragee et al., 2003; Carragee et al., 2006).

Studies have reported a reoccurrence of symptomatic herniated discs in 7%–18% of patients within 2 years of surgery (Carragee et al., 2006), and the hernia exceeds 6 mm in 27% of patients (Carragee et al., 2003). Successfully closing the rupture in the AF reduces the release of inflammatory factors from the disc and reduces the stimulation of nerve roots (Ren et al., 2020).

A number of methods have been proposed for repairing annular ruptures, including sutures, bioglues, tissue-engineered scaffolds, annular closure devices and AF patches (Chu et al., 2018). Sutures can reduce the need for subsequent surgery due to re-protrusion, but large ruptures often cannot be adequately repaired with sutures (Bateman et al., 2016). Bioglues have a short gelation time to rapidly close the defect and offer good biocompatibility, with high pressure retention capabilities (Scheibler et al., 2018). However, this technique is most effective with small ruptures, and its safety needs to be further investigated. Tissue-engineered scaffolds aim to restore the structural and mechanical environment of the AF while supporting cell growth, with good results being reported through *in vitro* and cellular experiments. However, implant dislocation is still a concern due to the difficulty of fixation (Bron et al., 2010). Annular closure devices lower the risk of recurrent herniation and reoperation, but complications such as endplate bone resorption have been reported, as well as longer procedure times and other device-related problems (Choy et al., 2018; Thomé et al., 2018). Alternatively, AF patches can provide physical occlusion of small or large AF ruptures with the occlusion component blocking the nuclear pulposus from exiting the disc. AF patches have gained wider acceptance in recent years with the use of biomaterials that provide excellent biocompatibility (Dewle et al., 2021; Borem et al., 2022). Polyurethane and porcine pericardium have been shown to mitigate inflammatory responses from the damaged AF while simultaneously plugging the rupture (McGuire et al., 2017; Bhunia et al., 2018). However, few studies have detailed how AF patches are secured to the ruptured annulus or their impact on the mechanical environment and lumbar kinematics.

Borem et al. developed a mechanically robust outer AF patch for repairing damaged intervertebral discs and demonstrated the capability to support cell activity (Borem et al., 2022). However, Borem also reported that the sutures broke and the patch ruptured after implantation, which is likely because such exterior repairs cannot withstand high intradiscal tensile forces (Bron et al., 2009). Also, when the spine is compressed, exterior patches may protrude and compress the posterior nerves in the back. Hegewald et al. concluded that AF implants should be placed on the inner region of the annulus, which would allow some bulging into the rupture without compromising the spinal canal (Hegewald et al., 2015). At the same time, this method can promote healing of the inner annulus and reduce the risk of implant dislocation (Hegewald et al., 2015). The natural healing process of AF ruptures starts on the outside and advances towards the center, but rarely surpasses the outer third of the rupture, leaving the inner regions of the AF unrepaired (Hegewald et al., 2015), which results in a high rate of *in situ* re-projection.

An effective implant for repairing a ruptured AF should be able to survive in the mechanical environment of the damaged IVD and allow for eventual integration into the surrounding tissue or promote the regeneration of healthy AF tissue. To date, few implants have effectively demonstrated this capability, especially implants that repair the inner surface of the annulus. Finite element model (FEM) analysis allows for one input factor such as the force or moment to be altered while keeping other factors constant. This is a convenient and inexpensive method of simulating different physiological conditions and has been widely used for assessing lumbar implants (Yao et al., 2006; Huang et al., 2022).

This pilot study uses a FEM of the lumbar spine to simulate the repair of a large box-shaped AF rupture using an AF patch placed on the inner surface of the annulus. The material properties and geometry of the patch were altered to determine the most stable combination to improve the primary stability and strength of the repaired tissue.

## 2 Materials and methods

### 2.1 Development of intact lumbar FEM

**FIGURE 1:**
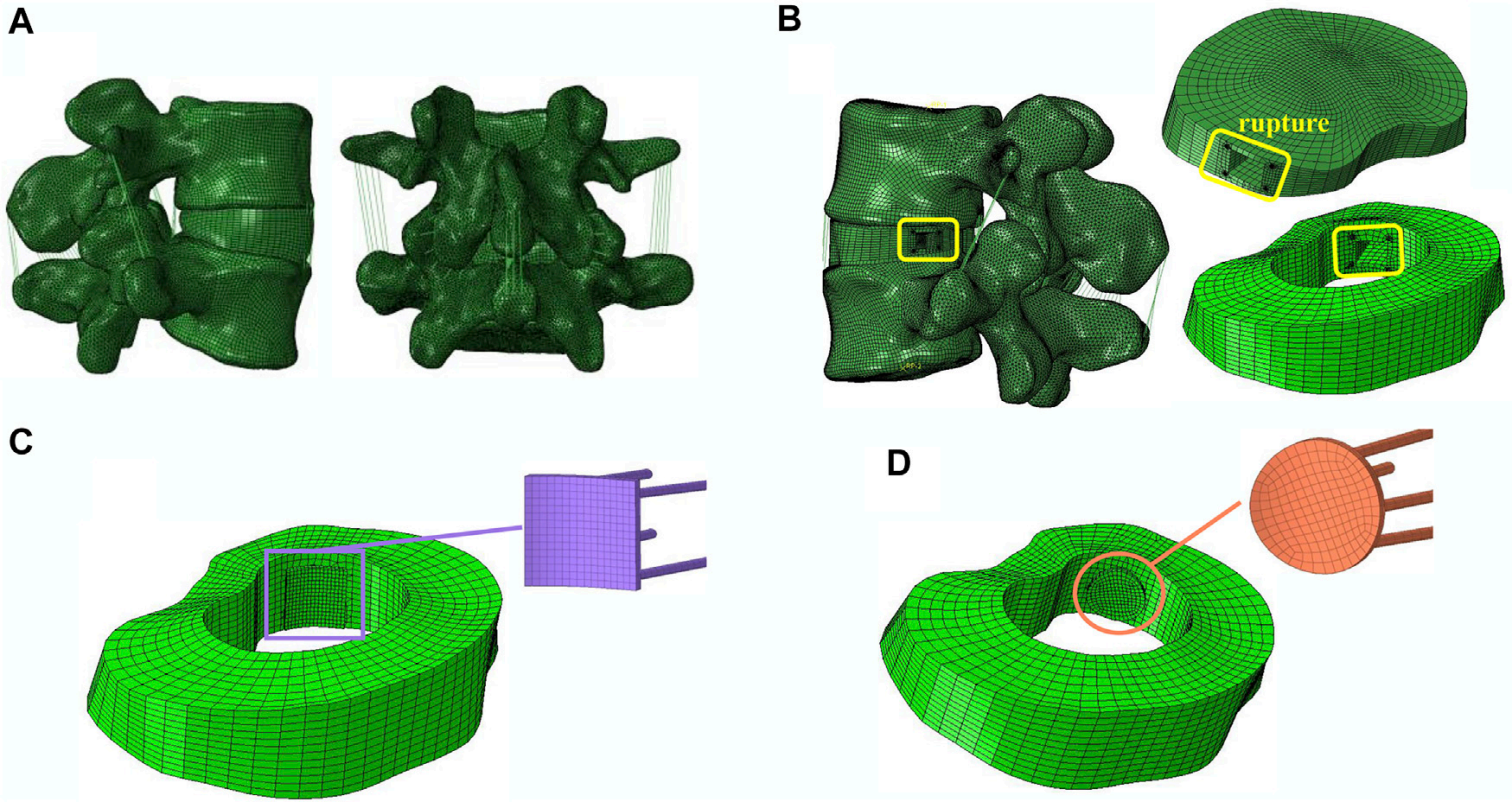
FEM of L4-L5 lumbar spine. **(A)** Intact Lumbar FEM. **(B)** Surgical Lumbar FEM. **(C)** Placement of a square patch **(D)**, or a circular patch on the inner side of the AF.

Figure 1 shows the 3-dimensional lumbar model used in this study, which was modified from a model previously developed by the research team (L4-L5) (Huang et al., 2022). Some studies report the non-homogeneous property of vertebrae, but other studies also report the reliability of vertebrae being isotropic (Hu et al., 2019). Since our future experiments require discussing the patch’s shape and material applied which is rather complicated, the vertebrae were simplified as isotropic and elastic using the elastic modulus and Poisson’s ratio (See Table 1 for specific values). The purpose of this study was to investigate the feasibility of repairing a ruptured AF with an inner patch, so the material properties of AF and NP were modeled as isotropic, incompressible, and hyperelastic using the Mooney-Rivlin equation (Schmidt et al., 2007; Aour and Damba, 2014) which can accurately mimic the behavior of the intervertebral disc.

**TABLE 1:** Properties of different components in the lumbar spine model.

Components	Young’s modulus (MPa)	Poisson’s ratio	Cross sectional area (mm)
Cortical bone	12,000	0.3	
Cancellous bone	100	0.2	
Posterior bone	3,500	0.25	
Endplate	500	0.3	
Nucleus pulposus	healthy disc: C1 = 0.12, C2 = 0.03 mildly herniated disc: C1 = 0.14, C2 = 0.035 severely herniated disc: C1 = 0.19, C2 = 0.045	0.45	
Annulus fibrosus	C1 = 0.18, C2 = 0.045	0.49	
Annulus fibers layers	360-550	—	0.76
ALL	15.6–20	0.3	63.7
PLL	10–20	0.3	18
LF	13–19.5	0.3	40
CL	7.5–33	0.3	32
ITL	12.0–58.7	0.3	1.8
ISL	8.8–15	0.3	25.2
SSL	9.8–12	0.3	35.1
Screws-rod system	1,10,000	0.3	

The intervertebral height of the model in this study was 9.90 mm, the vertebral body height of L4 was 31.06 mm, and the vertebral body height of L5 was 28.56 mm, all of which are within the ranges reported in literature (Campbell-Kyureghyan, 2005). Mesh convergence testing of the intact lumbar model was performed in Abaqus 2021 (Dassault Systemes Simulia Inc., France), and the resulting model consisted of 604,487 elements of 1 mm in size.

To validate the model, the L4-L5 intervertebral disc was subjected to pure compression of 300, 400, 500, 1,000, 1,600 and 2000 N to simulate the forces in different experiments, and the resulting NP pressure, vertical displacement, AF stress and disc bulging were compared against *in vitro* experimental data (Virgin, 1951; Gay et al., 2006; Yao et al., 2006; Huang et al., 2015; Actis et al., 2017; Zeng et al., 2017; Bashkuev et al., 2018). The model was also subjected to moments of 3.0, 7.5 and 8 Nm) to simulate flexion extension, lateral bending and axial rotation. The range of motion (ROM) of L4-L5 was compared with *in vivo* experimental results (Goel et al., 1985; Schilling et al., 2011; Kelly and Bennett, 2013).

### 2.2 Development of surgical lumbar FEM

Three disc conditions were assessed in this study, namely a healthy disc, a mildly herniated disc and a severely herniated disc. It has been reported that the elastic modulus of NP gradually increases with the size of the herniation, but there is little change in AF (Huang et al., 2015). Therefore, in this study, different severities of disc herniation were simulated by changing the elastic modulus of the NP. To validate the degenerative lumbar FEM, a torque of 10 Nm and axial compression force of 400 N were applied to the lumbar spine and the ROM was compared against results reported by (Mimura et al., 1994).

When an axial compression force of 400 N was applied to the intact model, the peak stresses were found to be concentrated in the left posterior-lateral region of the AF. Therefore, when simulating the disc herniation, the injury to the annulus was positioned in the left posterolateral region. This is consistent with reports that the posterolateral AF is at greatest risk of herniation (Marchand and Ahmed, 1990; Divi et al., 2022). A typical blade used for discectomy has a width of 5 mm (Fu et al., 2016), so in order to simulate the incision, a 5 mm × 5 mm box-shaped rupture was made in the left posterolateral region of the AF (Douglas et al., 2017; Ren et al., 2020). A patch with an area of 36 mm^2^ and thickness of 0.7 mm was attached to the inner wall of the AF to cover the rupture. The patches were slightly larger than the rupture and did not contact the endplate, which allowed for good closure while avoiding damage to the endplate. Four USP coding 3-0 Ethibond sutures with a diameter of 0.2 mm and Young’s modulus of 4.3 GPa were used to fix the patch corners to the AF (Chiang et al., 2012). The sutures used in the study were those commonly used in clinical practice. The material of the suture is referenced from ETHICON (ETHICON EXCEL^®^, United States), and the official website shows that the material is ethylene terephthalate. The interface between the patch and surrounding tissue was simulated with surface-to-surface contact elements. The friction coefficient between the patch and surrounding tissue was 0.1 (Pierrepont et al., 2018), and between the suture and AF was 0.1 (Cross et al., 2022). The disc height after repair with the patch is the same as that of the healthy model, which simulates the intraoperative implantation of an NP analogue that restores intervertebral height.

### 2.3 Material properties and geometry of the inner patch

The inferior endplate of the lower vertebra was rigidly fixed. A pure moment of 3 Nm was applied to the superior endplate of the upper vertebrae (Goel et al., 1985), allowing for six physiological movements to be simulated: flexion, extension, left bending, right bending, left rotation and right rotation. An axial compression force of 400 N was applied to the upper surface of the L4 –L5 lumbar motion segment to simulate the upper body weight of a 50 kg person (Yao et al., 2006).

The technique of repairing the inner aspect of the annulus fibrosus (AF) is difficult, and the smaller the size of the patch, the easier the operation. At the same time, the patch must be able to completely plug a 5 mm × 5 mm square rupture. Round and square patches are more suitable for plugging square ruptures, which have a greater effective area utilization. Other shapes, such as triangles and rectangles, require a larger volume of patch to repair the same size rupture. Considering the technical feasibility, this study repaired the AF rupture using a square patch and circular patch secured to the inner surface of the and recorded the pressure on the nucleus pulposus, vertical displacement, disc bulge, contact pressure on the cleft surfaces (CPCS), vertebral ROM and the maximum stress on the AF, patch and suture. The size of the disc bulge was defined as the maximum radial displacement of AF after compression. The CPCS is the maximum stress on the walls of the four sides of the AF rupture. A square patch was chosen because this was the design used in previous studies, which allows for better comparison (Dewle et al., 2021), and box-shaped ruptures can be more easily repaired using a similarly-shaped patch. The circular patch had the same area as the square patch and was used for comparison to determine which shape provided a better repair. The simulation was performed with different elastic moduli for the patch, ranging from 0 to 50 MPa (Yao et al., 2006).

## 3 Results

### 3.1 Validation of the intact and surgical lumbar FEM

**FIGURE 2:**
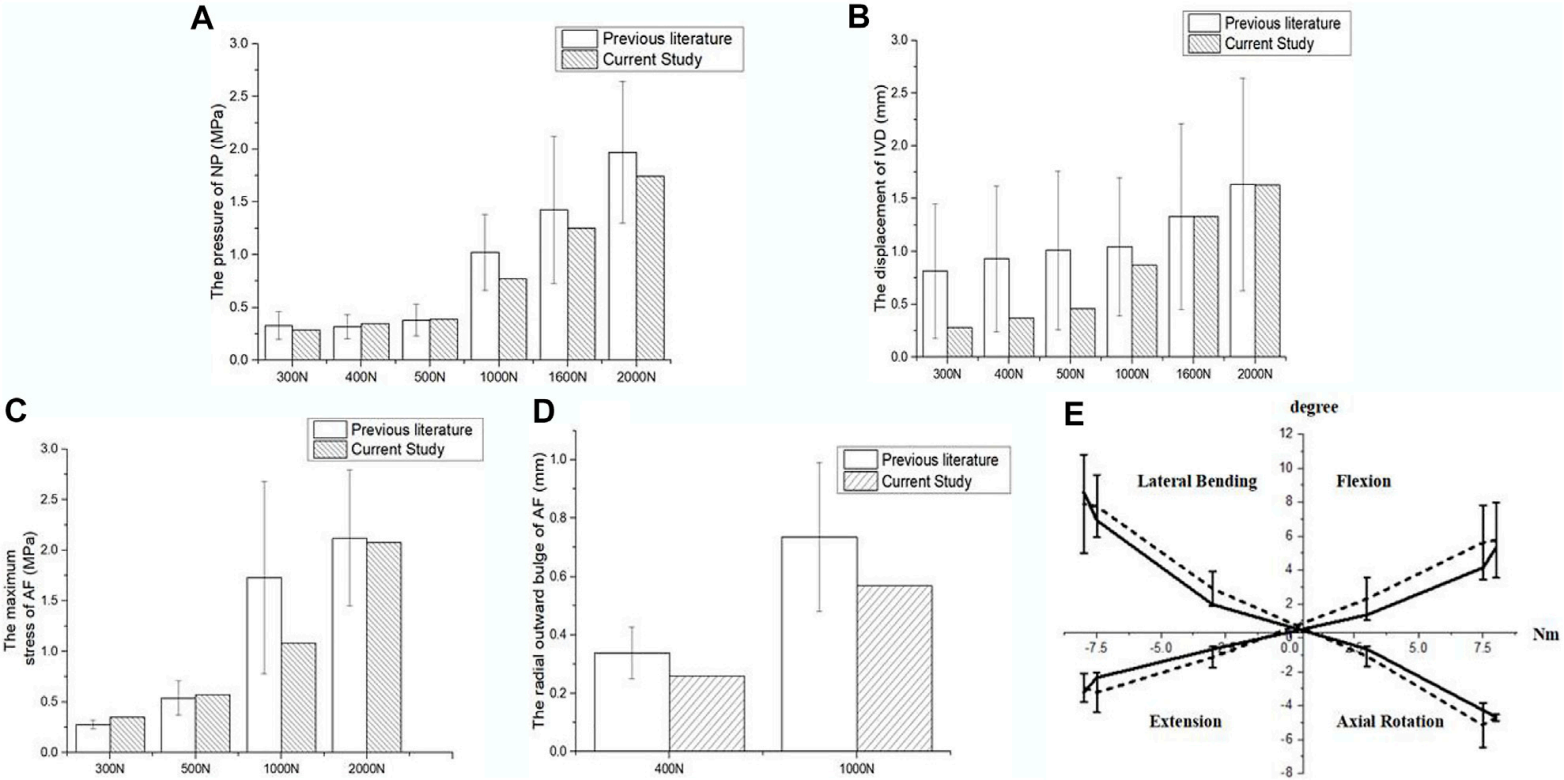
Validation of the intact lumbar FEM. Comparison of **(A)** NP pressure, **(B)** vertical displacement, **(C)** maximal AF stress, **(D)** disc bulge and **(E)** ROM of L4-L5 lumbar spine and *in vitro* experimental data.

As shown in Figure 2, the mean NP pressure and vertical displacement at the L4-L5 segment under axial compression forces of 300, 400, 500, 1,000, 1,600 and 2000 N was within the range of experimental data (Huang et al., 2015; Zeng et al., 2017; Bashkuev et al., 2018). The maximal AF stress under an axial compression of 300, 500, 1,000 and 2000 N was also in agreement with previous studies (Gay et al., 2006; Przybyla et al., 2006; Actis et al., 2017). The disc bulge under axial compression forces of 400 and 1000 N corresponded with results reported by Virgin and Yao et al. (Virgin, 1951; Yao et al., 2006), and the segmental ROMs under moments of 3, 7.5, and 8 Nm were also comparable to experimental results from literature (Figure 2E) (Goel et al., 1985; Schilling et al., 2011; Kelly and Bennett, 2013).

**FIGURE 3:**
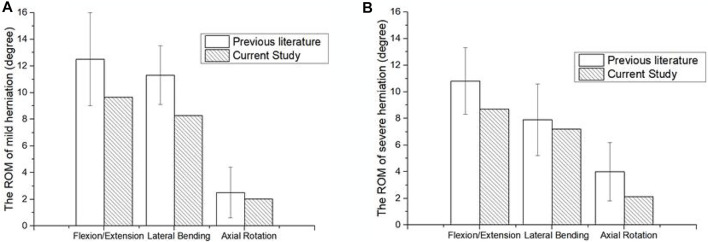
Comparison of surgical lumbar FEM to Mimura et al. (1994). **(A)** ROM of the mildly herniated model, and **(B)** the severely herniated model.

The ROM of the herniated lumbar FEM was comparable to experimental results from Mimura et al. when placed under a torque of 10 Nm and axial compression force of 400 N (Mimura et al., 1994). The good agreement between the results verified the accuracy of the mild and severely herniated models (Figure 3).

### 3.2 Effect of patch material properties on biomechanical response

**FIGURE 4:**
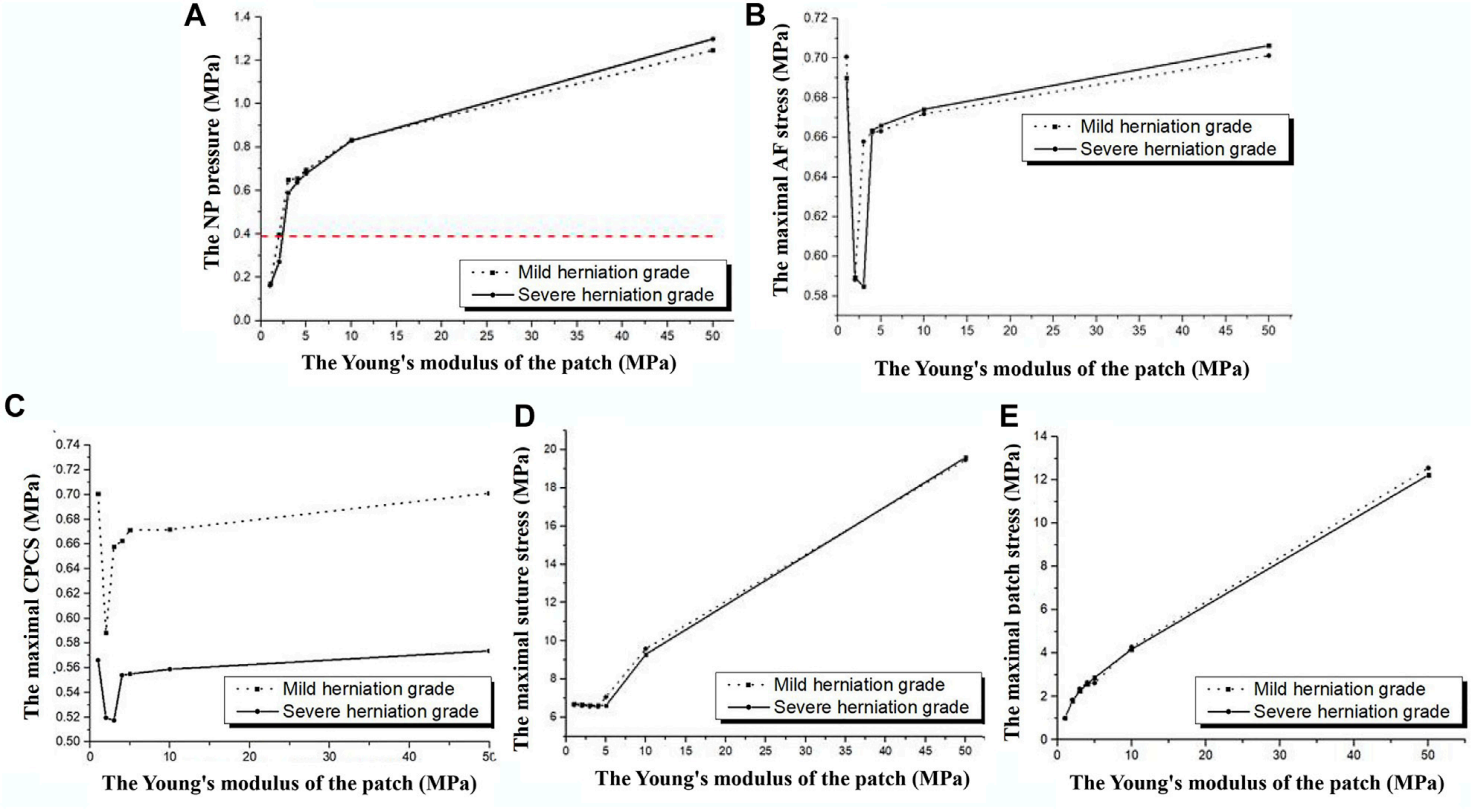
Model parameters in response to different Young’s modulus for the patch when placed under an axial compression of 400 N. **(A)** NP pressure, **(B)** maximal AF stress, **(C)** contact pressure on the cleft surfaces, **(D)** maximal suture stress and **(E)** maximal patch stress.

Figure 4 shows the model parameters after repairing the AF rupture with a square patch placed on the inner surface of the annulus. The model was loaded with an axial compression of 400 N. As shown in Figure 4A, the NP pressure in the intact model was 0.388 MPa (the red dotted line in Figure 4A) but increased rapidly in the repair model as the Young’s modulus of the patch increased, especially from 0 to 10 MPa. The NP pressure was closest to that of the intact model when the Young’s modulus of the patch was 2 MPa. All parameters were similar between the mild and severely herniated models, except for the maximum CPCS which showed a similar trend but a noticeable difference in values.

The maximum AF stress for the intact model was 0.464 MPa, but was exceeded by both repair models. As shown in Figure 4B, the minimum AF stress in the mildly degenerated model occurred with a Young’s modulus of 2 MPa for the patch (0.588 MPa), and the minimum value for the severely herniated model occurred with a Young’s modulus of 3 MPa for the patch (0.583 MPa).

The CPCS and maximum suture stress were at their lowest when the Young’s modulus of the patch was 2 MPa, with minimum values 0.587 and 6.574 MPa, respectively. The maximum stress on the patch increased with the Young’s modulus (Figure 4E). The NP pressure, maximum AF stress, maximum suture stress, maximum patch stress, and the CPCS were slightly greater in the severely degenerated model than the mildly degenerated model, but the values showed a similar trend.

**FIGURE 5:**
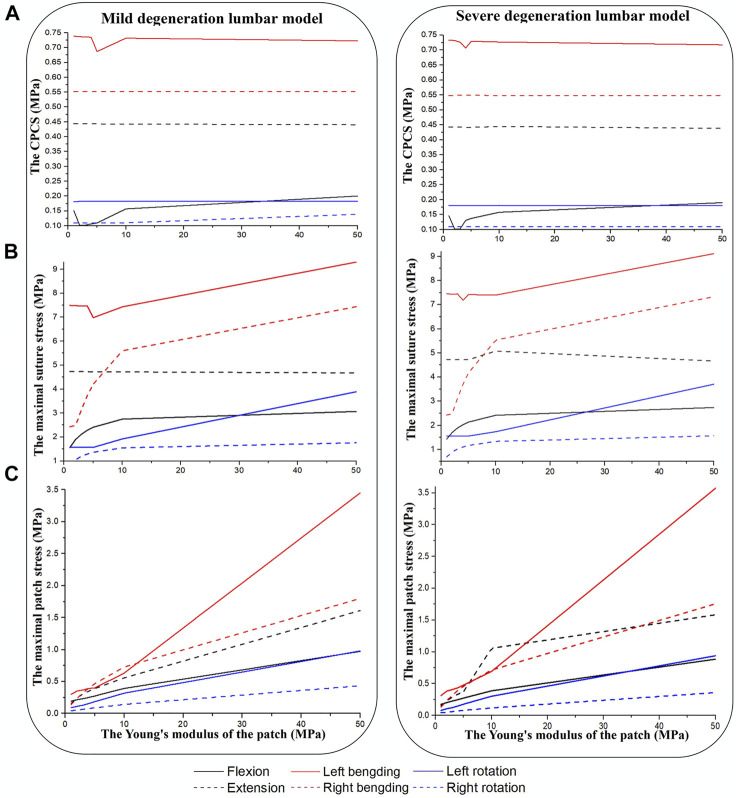
Results from herniated models when placed under a torque of 3 Nm. **(A)** CPCS, **(B)** maximal suture stress, **(C)** maximal patch stress.

Figure 5 shows results after repairing the AF rupture with a square patch secured to the inner surface of the annulus and a torque of 3 Nm applied to the spinal segment to simulate flexion, extension, bending and rotation. In flexion and left lateral bending, the CPCS was smallest when the Young’s modulus of the patch was 2 MPa (mildly herniated model) and 3 MPa (severely herniated model). There was no noticeable difference in CPCS between models with different Young’s moduli for the patch when placed in extension, right lateral bending and axial rotation (Figure 5A). The maximum suture stress increased with the modulus of the patch during other motions (Figure 5B). Similarly, the maximum patch stress also increased with increasing Young’s modulus in all motions (Figure 5C), but there was no significant difference between the herniated models. The results also showed that the lumbar mobility (ROM), vertical displacement and disc bulge in the repaired models were comparable to the intact model. Moreover, Changing the Young’s modulus of the patch did not have any significant effect on these parameters.

### 3.3 Effects of patch geometry on biomechanical properties

To assess how the geometry of the patch may affect the success of the repair, the modulus of the patch was set as 2 MPa and an axial compression force of 400 N and torque of 3 Nm were applied to the upper surface of the L4.

**FIGURE 6:**
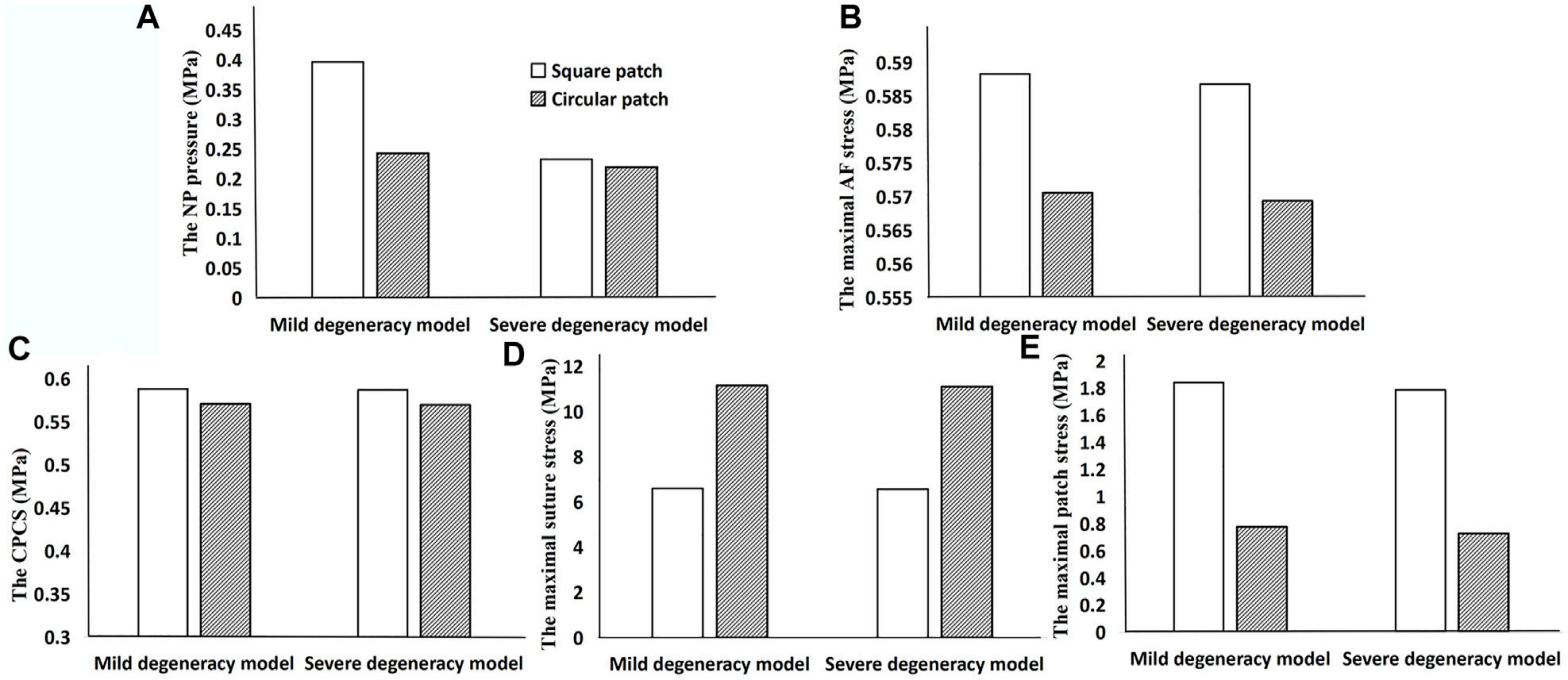
Results when using a square patch and circular patch to repair the ruptured annulus. **(A)** NP pressure, **(B)** maximal AF stress, **(C)** CPCS, **(D)** maximal suture stress, and **(E)** maximal patch stress.

**FIGURE 7:**
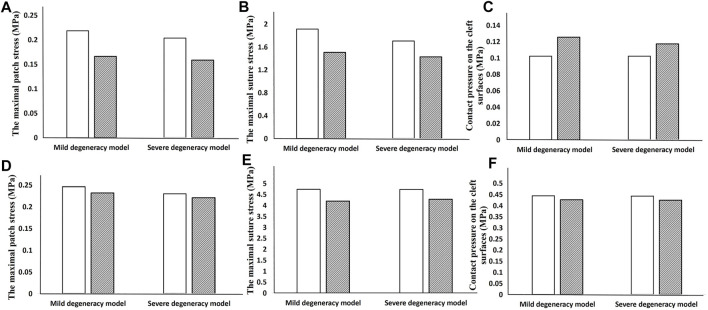
Results showing how the geometry of the patch affects the biomechanical performance when the lumbar segment is placed under a torque of 3 Nm. The top row is the result in flexion **(A–C)** and the bottom row is in extension **(D–F)**.

When the lumbar segment was placed under an axial compression force of 400 N, the circular patch showed a lower NP pressure, CPCS and patch stress than a square patch. However, the suture stress was greater. The degree of degeneration had a considerable effect on the NP pressure when using a square patch, but the circular patch was able to maintain a relatively constant pressure regardless of the severity of the herniation (Figure 6). In flexion and extension, using a circular patch resulted in lower stress on the patch and suture, but a larger CPCS in flexion (Figure 7).

**FIGURE 8:**
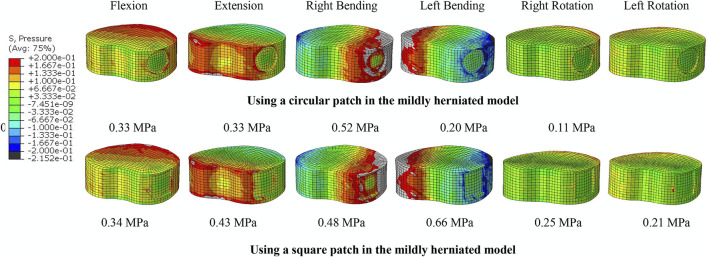
NP pressure when using a circular and square patch in the mildly herniated model under different motion conditions. The peak pressure is noted beneath each image.

There was no significant difference in the ROM or compressive stiffness of the lumbar spine after repairing the ruptured annulus with either a square patch or circular patch. However, the NP pressure was significantly less in all motions when using a circular patch (Figure 8).

## 4 Discussion

As a novel technique, annular repair is attracting more attention as an effective method of treating a ruptured disc while retaining the physiological environment. This study aimed to assess the feasibility of placing a patch on the inner surface of the AF to seal a ruptured disc using a validated finite element model. This technique is designed to reduce the loss of NP tissue and prevent re-leakage. The results showed that a circular patch with a Young’s modulus of 2–3 MPa applied to the inner surface of the AF region could immediately close the rupture and maintain a low NP pressure and restore the lumbar stability and stress distribution similar to the intact model.

A meta-analysis by Miller et al. reported that the risk of re-herniation and reoperation after lumbar discectomy was approximately threefold higher in patients with large versus small annular ruptures (Miller et al., 2018). There is no uniform clinical definition or any specific parameter of a large rupture. Guterl refered to ruptures larger than 3 mm as large ruptures (Guterl et al., 2013), while Carragee considered ruptures larger than 6 mm as large ruptures (Carragee et al., 2003). The 5 mm × 5 mm box-shaped rupture in the manuscript is a large rupture, which is between 3 and 6 mm. The inner AF patch introduced in this study is small and light enough to be secured to the AF but is also large enough to plug the rupture and provide a framework for the proliferation of fibroblasts, making it suitable for large annular ruptures. In patients with a fibrotic NP, we repair NP while removing the protrusion for decompression to provide enough space to place a patch, which is common in degenerating discs. The surgical approach should cause minimal disruption to the physiological environment, particularly since the primary function of the IVD is to maintain the stability and flexibility of the spine, which can usually be defined by the ROM and compressive stiffness (Rustenburg et al., 2019). In this study, repairing the ruptured disc with an inner AF patch restored the ROM and compressive stiffness of the segment similar to that of the healthy model, verifying that this technique can effectively seal the defect and maintain lumbar spine mobility.

To determine the most suitable material properties for the patch, different Young’s moduli were evaluated (up to a maximum of 50 MPa (Yao et al., 2006)) in terms of its effect on the NP pressure, vertical displacement, disc bulge, maximal AF stress and contact pressure on the cleft. Using a square patch, the NP pressure was found to increase with the patch modulus. The maximum NP pressure was 1.25 MPa (patch modulus of 50 MPa) in compression, which is well below the maximum allowable disc pressure (2.2 MPa) (Ahlgren et al., 2000). But this value is only slightly less than the maximum values found for the rupture intradiscal pressure (1.3 MPa) reported in the previous study (Iencean, 2000). Therefore, it is not recommended to choose a patch with a large modulus. The NP pressure in the repaired model was closest to the intact model when using a patch modulus of 2–3 MPa. In addition, using a circular patch resulted in a lower NP pressure than a square patch, which is less irritating to the AF and more conducive to AF repair. Consequently, a circular patch with a Young’s modulus of approximately 2–3 MPa should be used for repairing a herniated AF since it provides a similar NP pressure to the intact disc.

The loss of intervertebral height after repairing with the inner patch was less than that reported for tissue-engineered discs (0.34 vs. 0.48 mm) (Yao et al., 2006). Maintaining intervertebral height is important for regulating pressure within the disc because an increase in intradiscal pressure can cause the disc to bulge (Iencean, 2000). Discectomy may change the direction of motion in the inner AF layer on the posterior side of the disc, which is reflected by the inward bulging of the annulus. Inward bulging can increase shear stress between the AF and the end plate (Meakin et al., 2001), which can potentially lead to AF rupture and further herniation. Inward bulging of the internal AF was not found in this study, and the overall disc bulge was small and unrelated to the modulus of the patch. This indicates that the inner patch repair technique can maintain the stability and flexibility of the disc while minimizing the risk of nerve compression. For the intact disc, due to internal pressure, the maximal von Mises AF stress was located at the left posterior-lateral AF, which is the main reason the herniation was simulated here in the surgical model for this study. The maximum stress increases significantly after the AF is disrupted, which increases the risk of further AF degeneration. Previous studies also found that the rate of AF degeneration is much higher in patients with disc herniation than in normal subjects (O'Connell et al., 2011). AF stress changes are noticeable with a patch modulus of 1–5 MPa and there is little variation in stress once the patch modulus exceeds 5 MPa. Repairing the AF with a 2 MPa patch resulted in low AF stress, which is preferred. Biomechanically, the repair should generate adequate tension to withstand the hydrostatic pressure from the NP (Bron et al., 2010). Therefore, contact pressure between the annulotomy surfaces should be sufficient to overcome the intradiscal pressure, otherwise the annulotomy may leak. The results showed that the CPCS was greater than the NP pressure after patch repair. Unlike previous studies, increasing the modulus of the patch did not reduce the pressure on the contact cleft in a meaningful way, which may be due to the patch being too small to share the pressure on the contact cleft.

This study also considered the stress distribution on the repair patch and assessed the risk of failure. The patch must be able to withstand the maximum stress conveyed from the NP and inner region of the AF under different physiological loading conditions. The results predicted a maximum stress on the patch of 1.84 MPa, so the strength of the patch should exceed 1.84 MPa. The polypropylene sutures have an ultimate tensile strength of 23.15 ± 2.87 MPa (Türker et al., 2012). The maximum stress values on the suture (11.15 MPa) in the finite element model were much smaller than its ultimate tensile strength. It was found that the stress on the suture was greater when the repair was made with a circular patch of 2 MPa under axial compression, confirming the best hooping effect (Chiang et al., 2011).

From an in-vivo animal study, Chiang et al. showed fewer degenerative changes occur in repaired disks than unrepaired disks (Chiang et al., 2011). This current study confirmed that repairing the AF with an inner patch maintains good intervertebral height and lumbar stability. Based on previous experience with new implant development, stiffer implants often increase the maximum stress on the endplate, which may lead to endplate destruction (Schmidt et al., 2007), and soft implants may have difficulty restoring mechanical strength to the AF. Therefore, this study assessed the stress patterns when using patches with different modulus of elasticity. The results after repairing the AF with a 2–3 MPa circular patch were closest to the performance of the intact lumbar model. This can provide good initial stability and be expected to improve the long-term survivorship of the implant. Hydrogels with elastic modulus of 0.014–11 MPa have been developed by researchers (Dai et al., 2020; Zhang et al., 2021). The AF patch with 2–3 MPa can be prepared by adjusting the ratio of hydrogel components.

There are some limitations to this study. First, this was a preliminarily assessment of the reliability of AF repair with a medial patch which focused on the most suitable material properties for the device. Further studies, such as *in vitro*, animal models, are needed for product development to determine more details. Second, this study did not consider the implantation method for the patch, which is currently under investigation by the authors. Future studies may introduce custom surgical instrumentation, once it has first been confirmed that the patching technique is an effective method for repairing herniated IVDs. Third, the results of this study may only be applicable to cases where the AF rupture is located around the posterolateral aspect of the AF. This is the most common site of injury in the lumbar spine and so potentially is applicable in a large proportion of cases. Finally, the AF has a limited healing potential, and so whether the repair could generate clinically significant improvements over alternative methods requires further large-scale and comparative clinical studies.

## 5 Conclusion

This study verified the mechanical feasibility of repairing large annular ruptures with an inner patch and investigated how the material properties and geometry of the inner patch can influence the results. The results suggested that the AF inner patch should have a modulus in the range of 2–3 MPa and a strength greater than 1.84 MPa. In addition, the circular patch was better able to restore the properties of the IVD than the square patch. An inner patch with these characteristics can restore disc height and stress distribution. This study provides a basis for the design and development of related products in the future.

## Data Availability

The raw data supporting the conclusion of this article will be made available by the authors, without undue reservation.
